# Effect of Paris saponin I on radiosensitivity in a gefitinib-resistant lung adenocarcinoma cell line

**DOI:** 10.3892/ol.2014.2020

**Published:** 2014-04-01

**Authors:** HAO JIANG, PENGJUN ZHAO, JIANGUO FENG, DAN SU, SHENGLIN MA

**Affiliations:** 1 Department of Oncology, Zhejiang Hospital, Hangzhou, Zhejiang 310013, P.R. China; 2 Department of Radiation Oncology, Hangzhou Cancer Hospital, Hangzhou, Zhejiang 310002, P.R. China; 3 Department of Oncology, Zhejiang Cancer Hospital, Hangzhou, Zhejiang 310022, P.R. China; 4 Department of Oncology, Hangzhou First People’s Hospital, Hangzhou, Zhejiang 310006, P.R. China

**Keywords:** radiosensitivity, clonogenic cell survival, Paris saponin I, gefitinib resistance

## Abstract

Previous studies have observed that Paris saponin I (PSI) exerts a wide range of pharmacological activities, including cytotoxic activity against a number of malignancies, such as non-small cell lung cancers. The present study aimed to investigate the radiosensitization of PSI treatment on a gefitinib-resistant lung adenocarcinoma cell line, PC-9-ZD, and its possible mechanism. A 3-(4,5-dimethylthiazol-2-yl)-2,5-diphenyl-tetrazolium bromide assay was used to determine the growth inhibition effect of PSI. A clonogenic assay was performed to determine the radiosensitizing effect of PSI treatment on the PC-9-ZD cell line. A single-hit multi-target model was used to plot survival curves and calculate sensitizing enhancement ratios. The cell cycle was analyzed by flow cytometry and cell apoptosis was analyzed with fluorescein-isothiocyanate-Annexin V/propidium iodide and Hoechst staining. The expression levels of the proteins were detected by western blotting. There was a significant reduction observed in the proliferation of the PC-9-ZD cell lines that were treated with PSI. PSI enhanced the radiosensitivity of the PC-9-ZD cells with a sensitization enhancement ratio of 1.77. Furthermore, PSI induced G2/M arrest and apoptosis of the irradiated PC-9-ZD cells. Notably, B-cell lymphoma 2 (Bcl-2) was downregulated, and caspase-3, Bcl-2-like protein 4 (Bax) and cyclin-dependent kinase inhibitor 1 (P21^waf1/cip1^) were upregulated by the PSI treatment. The present study showed that PSI treatment exhibited potent radiosensitivity against gefitinib-resistant PC-9-ZD cells *in vitro*. This radiosensitivity was associated with cell cycle arrest at the G2/M phase, and apoptosis via an increase in caspase-3, Bax and P21^waf1/cip1^ as well as a decrease in Bcl-2 production.

## Introduction

Lung cancer has become one of the leading causes of cancer-related mortality worldwide and non-small cell lung cancer (NSCLC) represents 80% of lung cancers (1,2). The epidermal growth factor (EGFR) is a significant therapeutic target in NSCLC (3). Individuals with somatic mutations of the kinase domain of EGFR often respond to tyrosine kinase inhibitor (TKI) therapy, however, usually exhibit progressive disease following 6–8 months of therapy (4,5). Radiotherapy is extremely important for patients with NSCLC who are not eligible for surgery and patients that have experienced chemotherapy or TKI therapy failure. However, NSCLC cells are generally less sensitive to radiotherapy compared with SCLC cells, which results in radiotherapy failure (6). Although radiotherapy with increased irradiation dosage may delay tumor development, it leads to serious side-effects, including irradiation pneumonitis and a repressed hemopoietic system. Therefore, it is significant to use radiosensitizers to raise the therapeutic effect at a normal irradiation dosage. In the last few decades, increased attention has been focused on identifying biologically active cancer therapeutic agents derived from natural resources (7).

Rhizoma paridis is the root of *Paris polyphylla* Smith var. chinensis (Franch) Hara and *Paris polyphylla* Smith var. yunnanensis (Franch) Hand-Mazz. Preclinical studies have shown that Paris saponins (PS) have emerged as promising anticancer agents (8–12), and PSI exerts a wide range of pharmacological activities, including cytotoxic activity against certain malignancies, such as NSCLC (13–17). Therefore, PSI has been approved for cancer therapy due to its potential involvement in the suppression of tumor growth. However, PSI inhibition of signaling pathways and its radiosensitization in NSCLC-TKI resistance has not been identified. The present study focused on examining the radiosensitization effects of PSI on NSCLCs with acquired gefitinib resistance *in vitro* and to further verify the possible mechanisms.

## Materials and methods

### Drugs and reagents

**Figure 1:**
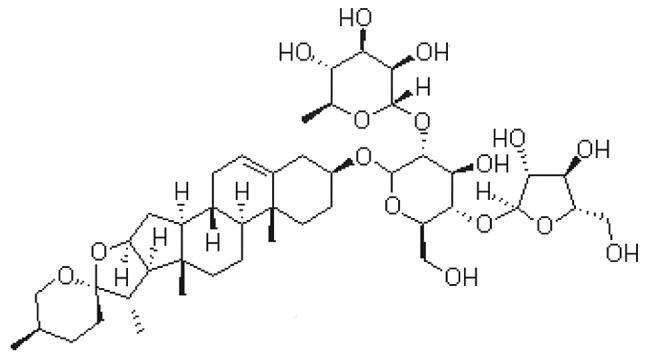
Chemical structure of Paris saponin I.

PSI (C_44_H_70_O_16_) was obtained from Zhejiang Meidikang Ltd. (Zhejiang, China) and the structure of the compound is shown in Fig. 1. PSI was prepared as a 20-mmol/l stock in dimethyl sulfoxide (DMSO) and stored at −20°C. PSI was diluted with cell culture medium to concentrations of 0.5, 1.0, 2.0, 3.0, 4.0, 5.0, 6.0, 7.0, 8.0 and 9.0 μg/ml, with a final DMSO concentration of 0.25% (v/v). Dulbecco’s modified Eagle’s medium (DMEM) was purchased from Gibco-BRL (Carlsbad, CA, USA), the Cycle Test^™^ Plus DNA reagent and Annexin V-FITC & Propidium Iodide (PI) Apoptosis Detection kits were obtained from Becton Dickinson and Co., (Franklin Lakes, NJ, USA), Hoechst 33258 was purchased from BYT Co., (Nanjing, China), mouse and rabbit antibodies against caspase-3, Bcl-2-like protein 4 (Bax), B-cell lymphoma 2 (Bcl-2) and cyclin-dependent kinase inhibitor 1 (P21^waf1/cip1^) were obtained from Cell Signaling Technology (Danvers, MA, USA) and β-actin from Santa Cruz Biotechnology, Inc., (Santa Cruz, CA, USA). This study was approved by the Ethics Committee of Zhejiang Hospital and was performed according to the Declaration of Helsinki. Written informed consent was obtained from the family of the patients.

### Cell culture

PC-9-ZD (18), an NSCLC cell line resistant to gefitinib following long-term exposure to the drug, was obtained from the Laboratory of Biochemistry and Molecular Biology, Tongji University (Shanghai, China). The PC-9-ZD cells were grown in DMEM supplemented with 10% fetal bovine serum, 100 μg/ml penicillin and 100 μg/ml streptomycin at 37°C in a 5% CO_2_ humidified atmosphere.

### Cell proliferation assay

The cell proliferation assays were performed using the MTT method, according to the manufacturer’s instructions. The cells were seeded in 96-well plates (Costar; Corning Life Sciences, Cambridge, MA, USA) with 5,000 cells/well. Subsequent to an overnight incubation, triplicate wells were treated with various concentrations of PSI for 24 h. Following this, 20-μl MTT solutions (5 mg/ml in phosphate-buffered saline; PBS) were added to each well and incubated for 4 h at 37°C. The MTT formazan was dissolved in 150 μl DMSO and the absorbance was measured with a microplate reader (Multiskan MK3; Thermo Labsystem, Waltham, MA, USA) at a wavelength of 570 nm. The drug-cell inhibition curve used the drug concentration as the abscissa axis and the inhibition ratio of the drug as the vertical axis. The 50% growth inhibition (IC_50_) was subsequently calculated according to the curve.

### Determination of cell radiosensitivity

The growing cells were exposed to PSI with a concentration of 20% of the IC_50_ for 3 h, and irradiated at 0, 1, 2, 4, 6, 8 or 10 Gy with a 6-MV X-ray. After 24 h, the cells were trypsinized, counted and seeded at various dilutions according to the irradiation dose and cultured for 14 days; the colonies were fixed, stained with crystal violet and counted. Only the colonies containing 50 cells were scored and the experiments were performed in triplicate. The cell-survival curve used the irradiation dosage as the abscissa axis and the survival fraction (SF) as the vertical axis. The average lethal dosage of cells (D_0_) and the quasi-field dosage (Dq), which indicates the repair ability of cells to sublethal injury, and extrapolation number (N) values were calculated according to the curve. The sensitization enhancement ratio (SER) was calculated according to the following equations: SF = 1-(1-exp[−D/D_0_])^N^; Dq = InN/(1/D_0_); and SER = control group D_0_ value/treatment group D_0_ value.

### Cell-cycle distribution

The experimental groups were the control, radiation and PSI + radiation groups. The radiation group received a 2-Gy treatment and the PSI + radiation group received a 2-Gy and a PSI treatment using a concentration that was 20% of the IC_50_. Cells were harvested at 12, 24 and 48 h and were fixed with 70% ethanol and stored overnight at −20°C. The cells were centrifuged using a Heraeus Labofuge 400 centrifuge (Thermo Fisher Scientific, Waltham, MA, USA) at 300 × g and washed twice with PBS. They were labeled with 50 mg/ml PI and protected from light for 30 min prior to analyses by flow cytometry that were conducted with a multi-cycle system software package (CellQuest version 3.1; Beckman Coulter, Inc., Brea, CA, USA). Experiments were performed in triplicate.

### Apoptosis measurement

Apoptosis was measured by PI/Annexin V double and Hoechst staining. The experimental groups were the control, radiation and the PSI + radiation groups. The radiation group received a 2-Gy treatment and the PSI + radiation group received a 2-Gy and PSI treatment using a concentration that was 20% of the IC_50_. The cells were harvested at 24 h following treatment and stained with PI and Annexin V. The apoptotic fraction was detected by flow cytometry (BD FACSCalibur; BD Biosciences, Franklin Lakes, NJ, US). The cells were washed in PBS, stained with Hoechst 33528 (5 μg/ml in PBS) for 15 min at room temperature and observed under a fluorescence microscope (Olympus BX-60, Olympus Optical Co., Ltd., Tokyo, Japan) equipped with 356-nm excitation and 492-nm emission band-pass filters.

### Western blot analysis

The cells were exposed to a PSI treatment of 20% of the IC_50_ for 3 h and subsequently irradiated at a dose of 2 Gy and incubated for 24 h. Total cell lysates were separated by sodium dodecyl sulfate-polyacrylamide gel electrophoresis and transferred to a polyvinylidene fluoride membrane (Seebio Biotech, Inc., Shanghai, China). The membranes were incubated overnight with primary antibodies [1:1,000; caspase-3 mouse monoclonal antibody (mAb), Bax rabbit mAb, Bcl-2 rabbit mAb and p21 Waf1/Clip1 rabbit mAb] at 4°C with gentle agitation (Wave-SI slim shaker; TAITEC Corporation, Koshigaya, Japan). The membranes were incubated for 2 h with a horseradish peroxidase-labeled secondary antibodies (1:2,000; affinity purified goat antimouse IgG and goat antirabbit IgG) at room temperature. All membranes were detected using the ECL system (Santa Cruz Biotechnology Inc.).

### Statistical analysis

The t-test (mean comparison in two samples) and single-factor variance analysis (mean comparison in multiple samples) were assessed using SPSS 17.0 (SPSS, Inc., Chicago, IL, USA) and experimental data are indicated by the mean ± SD P<0.05 was considered to indicate a statistically significant difference.

## Results

### PSI inhibits the proliferation of PC-9-ZD cells

**Figure 2:**
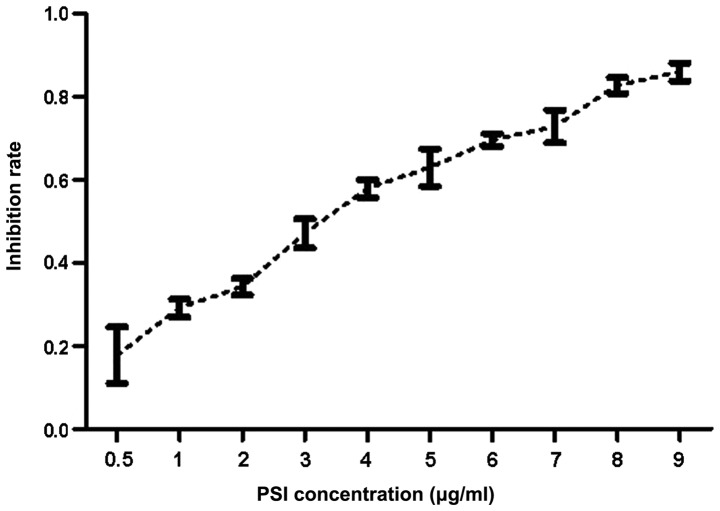
Inhibition rate of PSI on the PC-9-ZD cells. Percentage of cell viability was determined by MTT assay. PSI, Paris saponin I.

The MTT assay (Fig. 2) showed that PSI treatment inhibited cell proliferation in a dose-dependent manner. The concentration required to achieve IC_50_ was estimated to be 2.5132 μg/ml at 24 h.

### PSI enhances the radiosensitivity of PC-9-ZD cells

**Figure 3:**
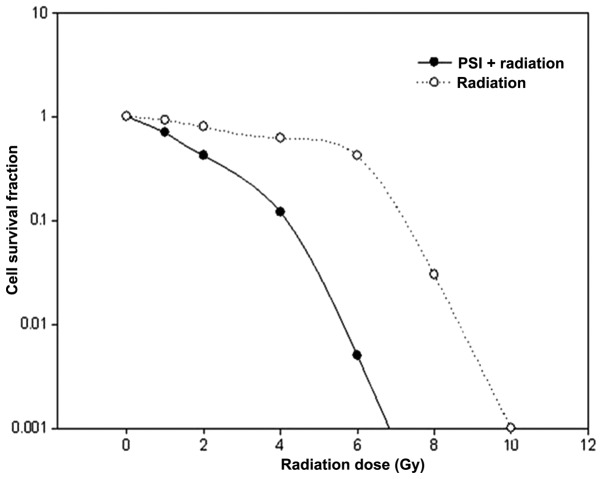
Clonogenic survival of PC-9-ZD cells. The cells treated with or without 0.5 μg/ml PSI were exposed to X-ray irradiation; range, 0–10 Gy. Subsequently, cells were incubated for 14 days and the number of colonies with >50 cells was scored. PSI, Paris saponin I.

0.5 μg/ml PSI and 20% of the IC_50_ served as the experiment concentration. A multi-target click mathematical model (Fig. 3) simulated the cell SF curve, through which an associated equation and radioactivity parameters, D_0_ and Dq (Table I) were obtained. The results show a declined SF2, a decreased Dq and the shoulder of the survival curve is decreased, with an SER value of 1.77, based on D_0_.

### PSI induces the G2/M arrest of irradiated PC-9-ZD cells

To identify whether the radiosensitivity of PSI was due to cell cycle arrest, the influence of PSI treatment (0.5 μg/ml; 20% of IC_50_) on cell cycle distribution was observed (Table II). The result showed that irradiation alone induced G2/M arrest in a time-dependent manner with an increased cell density at the G2/M phase from 16.56 to 24.07% compared with the control group (P<0.05). However, PSI altered the cycle distribution of irradiated cells significantly, leading to cell cycle arrest at the G2/M phase in a time-dependent manner with an increased cell density at the G2/M phase from 27.63 to 39.30% compared with the radiation group (P<0.01).

### PSI induces apoptosis of irradiated PC-9-ZD cells

**Figure 4:**
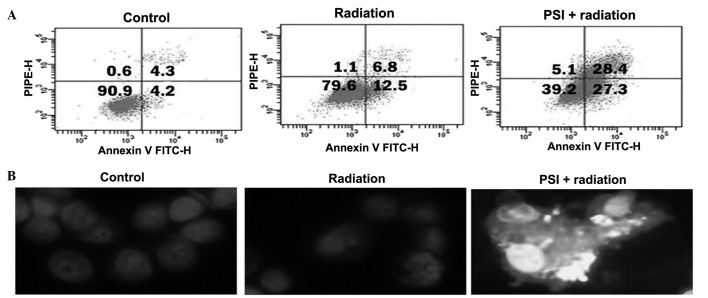
(A) PSI on the apoptosis of irradiated PC-9-ZD cells. The experimental groups were the control, radiation and PSI + radiation groups. The radiation group received 2-Gy treatment and the PSI + radiation group received 2-Gy and a PSI treatment of 20% of the IC_50_. Cells were harvested at 24 h following treatment and stained with propidium iodide and Annexin V. The apoptotic fraction was detected by flow cytometry. (B) Fluorescence imaging of the nuclei in the control, radiation and PSI + radiation groups by Hoechst 33258 staining. PC-9-ZD cells stained with Hoechst 33528 were visualized by fluorescence microscopy. Condensed and fragmented nuclei were observed in the PSI + radiation group, but not in the control and radiation groups. PSI, Paris saponin I.

In order to investigate the radiosensitivity mechanism of PSI, the influence of PSI (0.5 μg/ml; 20% of IC_50_) on cell apoptosis was observed by Annexin V/PI double and Hoechst staining assays. Irradiation increased apoptosis at 24 h, however, the PSI combination treatment further increased the apoptosis ratio up to the higher level (P<0.01; Fig. 4A). Fig. 4B demonstrates that the control and irradiated cells were morphologically normal, and the nuclei were regularly shaped and evenly stained. However, typical morphological changes of apoptosis, including nuclear shrinkage, DNA condensation and chromatin fragmentation were identified in the PSI + radiation group. This indicates that PSI treatment can further increase apoptosis that is induced by radioactive rays.

### PSI upregulates P21^waf1/cip1^, caspase-3 and Bax, and downregulates Bcl-2 protein expression of irradiated PC-9-ZD cells

**Figure 5:**
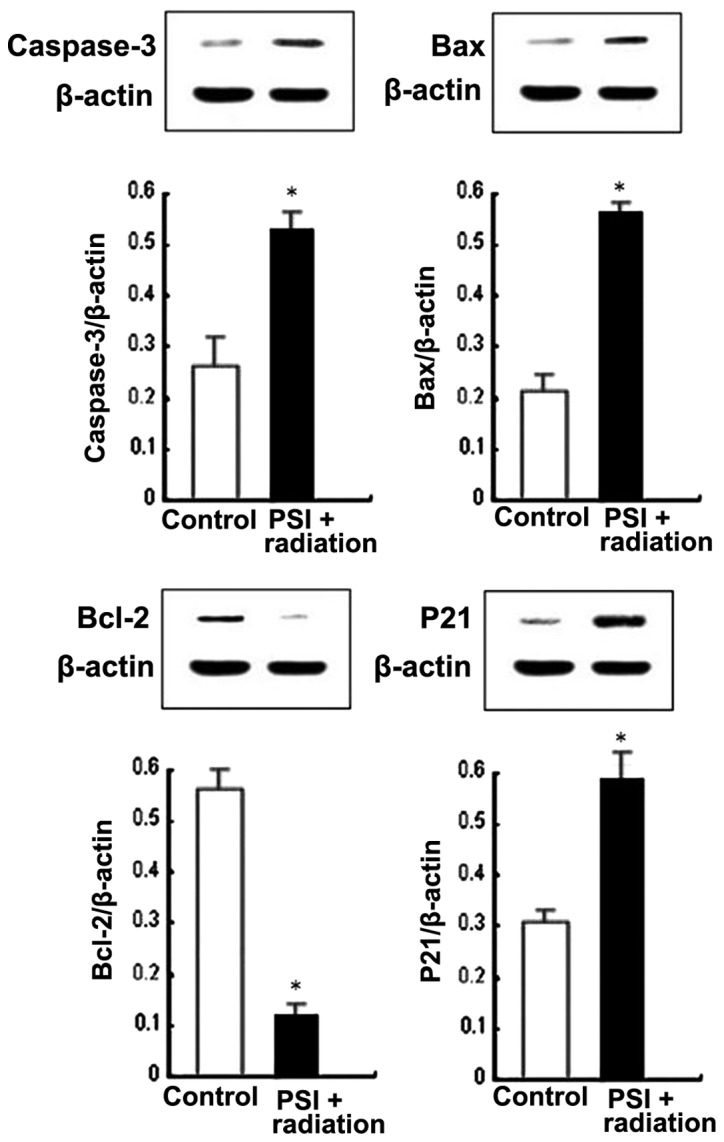
PSI significantly increases the expression of P21^waf1/cip1^, caspase-3 and Bax, however, decreases the expression of Bcl-2 of the irradiated PC-9-ZD cells. β-actin expression served as an internal control. Values are the means of triplicate analysis and error bars show standard deviations. ^*^P<0.05 vs. control group. Bax, Bcl-2-like protein 4; PSI, Paris saponin I; Bcl-2, B-cell lymphoma 2; P21^waf1/cip1^, cyclin-dependent kinase inhibitor 1.

In order to confirm which modulating molecules were involved in the PSI treatment on cell cycle arrest and apoptosis of irradiated PC-9-ZD cells, P21^waf1/cip1^, the most significant regulator in the cell cycle checkpoint, and caspase-3, Bax and Bcl-2, which are significant apoptosis regulators, were investigated. The results showed that PSI significantly increased the expression of P21^waf1/cip1^, caspase-3 and Bax in irradiated PC-9-ZD cells. Notably, PSI markedly decreased Bcl-2 expression in the irradiated cells (Fig. 5). This incidates that increased P21^waf1/cip1^ expression contributes to G2/M arrest, and increased caspase-3 and Bax expression levels; however, a decreased Bcl-2 expression contributes to apoptosis, which is induced by PSI treatment in irradiated cells.

## Discussion

In the present study, simulating the comparison for the irradiation-survival curve of PC-9-ZD cells using a multi-target click mathematical model formula reveals that the D_0_, Dq and N values of cells is decreased following a PSI treatment of irradiated cells. PSI enhances the radiosensitivity effect of PC-9-ZD cells with an SER of 1.77. These results indicate that, following treatment with PSI, the average lethal dosage of the PSI + radiation group is decreased compared with the control group, the shoulder is clearly decreased, and the repair ability of cell sublethal injury is markedly decreased. The radiosensitivity effect of PSI treatment is evidently increased in the PC-9-ZD cells, the average lethal dosage is decreased with irradiation, the decrease of shoulder is more significant and the repair ability of cell sublethal injury is clearly decreased.

Further studies have been conducted to investigate the radiosensitivity mechanism of PSI treatment. The results from assessing the cell cycle and apoptosis in the present study indicate that PSI predominantly induces G2/M phase arrest and apoptosis. Apoptosis was the primary reason for cell death induced by PSI in the irradiated cells. It was shown that a PSI combination treatment advanced the apoptosis ratio up to a higher level. In addition, PSI further increases apoptosis that is induced by radioactive rays. Caspases are essential mediators of apoptosis. Among them, caspase-3 is a frequently activated death protease, catalyzing the specific cleavage of numerous key cellular proteins (19). The Bcl-2 family, which comprises of anti-apoptotic (including Bcl-2 and Bcl-xl) and proapoptotic (including Bax and Bak) members, is the predominant controller and mediator of cell apoptosis (20,21). Particularly, the high Bcl-2/Bax ratio is considered to be a crucial factor of cell resistance to apoptosis (22,23). To investigate the role of PSI in the irradiation-induced apoptosis pathway in gefitinib-resistant PC-9-ZD cells, the Bcl-2 family proteins and the caspase-3 protein were analyzed in the present study. The results indicated that Bcl-2 was decreased, and Bax and caspase-3 were increased as a result of PSI treatment. Thus, PSI promotes the irradiation-induced apoptosis via the association between Bcl-2 and Bax, and caspase-3, eventually leading to enhanced radiosensitivity.

Furthermore cell cycle arrest was the major reason for cell death, which was induced by PSI in the irradiated cells. Cell cycle regulation was significant for cell proliferation and the cells exhibited varied radiosensitivity in various phases of the cell cycle. Cells were most sensitive to irradiation during the G2/M phase, less sensitive during G1, and least sensitive near the end of the S phase (24). It was shown in the present study that PSI treatment significantly altered the cycle distribution of the irradiated cells, leading to cell cycle arrest at the G2/M phase in a time-dependent manner, with an increased cell density at the G2/M phase from 27.63 to 39.30%. Previously, P21^waf1/cip1^ was considered to be the most significant cell cycle checkpoint protein (25–27). In the present study it was shown that PSI significantly increased the expression of P21^waf1/cip1^, which resulted in cell-cycle progression via G2/M arrest in the PC-9-ZD cells. This indicates that P21^waf1/cip1^ is significant in mediating cell growth through G2/M arrest in gefitinib-resistant cell lines.

In conclusion, PSI exhibited potent radiosensitivity against gefitinib-resistant PC-9-ZD cells *in vitro*. This radiosensitivity was associated with the cell cycle arrest at the G2/M phase and apoptosis via increased caspase-3, Bax and P21^waf1/cip1^ and decreased Bcl-2 production. Therefore, PSI may have the potential to be a radiosensitizer, however, this requires further investigation.

## Figures and Tables

**Table I:** Parameters of multi-target click mathematical model.

Group	D_0_	Dq	N	SF2 (%)	SER D_0_
Radiation	0.5105	0.1636	1.3777	37.23	-
PSI + radiation	0.2887	0.0598	1.2302	17.43	1.77

D_0_, average lethal dosage of cells; Dq, quasi-field dosage; N, extrapolation number; SF2, survival fraction 2; SER, sensitization enhancement ratio; PSI, Paris saponin I.

**Table II:** Effect of PSI on the G2/M phase of irradiated PC-9-ZD cells.

	Cell density (%, mean ± standard deviation)		
	
Group	12 h	24 h	48 h
Control	7.18±1.44	9.45±2.51	10.89±2.72
Radiation^a^	16.56±1.35	21.67±2.25	24.07±2.47
PSI + radiation^b^,^c^	27.63±2.16	35.88±2.14	39.30±2.53

a P<0.05 vs. control group;

b P<0.01 vs. control group;

c P<0.01 vs. radiation group.

PSI, Paris saponin I.
